# CRISPR/Cas9‐based functional analysis of yellow gene in the diamondback moth, *Plutella xylostella*

**DOI:** 10.1111/1744-7917.12870

**Published:** 2020-09-18

**Authors:** Yajun Wang, Yuping Huang, Xuejiao Xu, Zhaoxia Liu, Jianyu Li, Xue Zhan, Guang Yang, Minsheng You, Shijun You

**Affiliations:** ^1^ State Key Laboratory of Ecological Pest Control for Fujian‐Taiwan Crops Institute of Applied Ecology Fujian Agriculture and Forestry University Fuzhou 350002 China; ^2^ Joint International Research Laboratory of Ecological Pest Control Ministry of Education Fuzhou 350002 China; ^3^ Key Laboratory of Integrated Pest Management for Fujian‐Taiwan Crops Ministry of Agriculture Fuzhou 350002 China; ^4^ Department of Physiology & Neurobiology University of Connecticut Storrs CT 06269 USA; ^5^ Fujian Key Laboratory for Monitoring and Integrated Management of Crop Pests Institute of Plant Protection Fujian Academy of Agricultural Sciences Fuzhou 350013 China

**Keywords:** CRISPR/Cas9, diamondback moth, genetically based control, novel marker, yellow gene

## Abstract

The diamondback moth, *Plutella xylostella* (L.), is an economically important pest of cruciferous crops worldwide. This pest is notorious for rapid evolution of the resistance to different classes of insecticides, making it increasingly difficult to control. Genetics‐based control approaches, through manipulation of target genes, have been reported as promising supplements or alternatives to traditional methods of pest management. Here we identified a gene of pigmentation (yellow) in *P. xylostella*, *Pxyellow*, which encodes 1674 bp complementary DNA sequence with four exons and three introns. Using the clustered regularly interspersed palindromic repeats (CRISPR)/CRISPR‐associated protein 9 system, we knocked out *Pxyellow*, targeting two sites in Exon III, to generate 272 chimeric mutants (57% of the CRISPR‐treated individuals) with color‐changed phenotypes of the 1st to 3rd instar larvae, pupae, and adults, indicating that *Pxyellow* plays an essential role in the body pigmentation of *P. xylostella*. Fitness analysis revealed no significant difference in the oviposition of adults, the hatchability of eggs, and the weight of pupae between homozygous mutants and wildtypes, suggesting that *Pxyellow* is not directly involved in regulation of growth, development, or reproduction. This work advances our understanding of the genetic and insect science molecular basis for body pigmentation of *P. xylostella*, and opens a wide avenue for development of the genetically based pest control techniques using *Pxyellow* as a screening marker.

## Graphical abstract

We identified a gene of pigmentation (yellow) in P. xylostella, Pxyellow, and performed a clustered regularly interspersed palindromic repeats (CRISPR)/CRISPR‐associated protein 9 (Cas9)‐mediated knockout experiment to verify that *Pxyellow* plays an essential role in the body pigmentation without being directly involved in regulation of growth, development, and reproduction. Such findings prove the effectiveness of *Pxyellow* as a screening marker for genetically based pest control techniques.

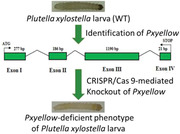

Dear Editor,

The diamondback moth (DBM), *Plutella xylostella* (L), is one of the most widely distributed lepidopteran pests all over the world, which causes great economical damage to cruciferous crops (Furlong *et al*., 2013). Due to the overuse and misuse of insecticidal chemicals, rapid evolution of resistance to all major classes of pesticides has made DBM increasingly difficult to be effectively controlled. Genetics‐based strategies have been proposed as environmentally friendly alternatives to the overuse of insecticides in pest management (Alphey, 2014). Recently, a novel genetic approach of self‐sustaining population elimination, clustered regularly interspersed palindromic repeats (CRISPR)‐based gene drive system, has been developed in the model insect *Drosophila melanogaster* (Gantz & Bier, 2015) as well as non‐*Drosophila* disease vectors (Gantz *et al*., 2015; Li *et al*., 2020), which all showed promising population control results. Although some sex‐determination genes have been proposed as potential targets for genetics‐based population suppression (Kyrou *et al*., 2018; Wang *et al*., 2019), in order to build gene‐driven prototypes and assess the driving efficiency in different species, it is desirable to target endogenous phenotypic genes, such as *yellow* (one of the main melanin synthesis pathway genes), in the first place. In *Drosophila*, yellow protein was required in producing black melanin, which maintained normal black body pigmentation (Wittkopp *et al*., 2002). Mutations in the *yellow* gene were reported to cause a change in the melanin synthesis pattern, turning the coloration from black to yellow (Wittkopp *et al*., 2002). Similar phenotypes derived from *yellow*‐deficient insects were also observed in *Tribolium castaneum* (Rylee *et al*., 2018) and *Bombyx mori* (Xia *et al*., 2006; Futahashi *et al*., 2008). In addition to yellowish body color, the disruption of *yellow* gene also led to a dehydration‐like phenotype during a short developmental stage in *Agrotis ipsilon* (Chen *et al*., 2018). However, the regulation of body pigmentation and the possible functions of *yellow* in DBM remain unclear.

CRISPR/CRISPR‐associated protein 9 (Cas9)‐induced mutagenesis of target genes have been documented in multiple species of moth insects, including *Spodoptera littoralis* (Koutroumpa *et al*., 2016), *Spodoptera litura* (Bi *et al*., 2019), *Helicoverpa armigera* (Wang *et al*., 2016; Khan *et al*., 2017), and *A. ipsilon* (Chen *et al*., 2018). Since 2016, using the CRISPR/Cas9 approach, several cases of gene manipulation in DBM have been reported by our team (Huang *et al*., 2016; Peng *et al*., 2019; Chen *et al*., 2020) and another research group (Wang *et al*., 2019), providing a relatively mature gene editing platform in this global pest. Therefore, as an efficient genome editing tool, the CRISPR/Cas9 system was utilized in this study to verify the functions of *yellow* gene in DBM (hereafter *Pxyellow*).

To identify the coding sequence of *Pxyellow*, we used one of the *yellow* genes in *B. mori*, *Bmyellow‐y* (NP_001037434.1), as a query to blast against our previously published *P. xylostella* genome sequence (You *et al*., 2013). Nine putative *yellow* homologs were found in *P. xylostella* (gene ID: *Px007091*, *Px007817*, *Px005439*, *Px016714*, *Px011025*, *Px015683*, *Px005437*, *Px005436* and *Px010416*). The deduced amino acid sequences of these genes contained the conserved domain MRJP (major royal jelly protein), which is the characteristic motif of yellow proteins across different insect species (e.g., *B. mori* and *D. melanogaster*), although proteins encoded by *Px005436* and *Px010416* only comprised partial MRJP domain (Table S1). Phylogenetic analysis showed that these genes were well clustered with other insect homologs, indicating the potentially conserved functions of these genes in different species (Fig. S1 and Table S2). Based on the well‐studied role of Yellow‐y protein in promoting melanization in *B. mori* (Futahashi *et al*., 2008) and *T. castaneum* (Arakane *et al*., 2010), the most likely *yellow‐y* ortholog in DBM (gene ID: *Px007091*), which showed the lowest E‐value by blasting *B. mori yellow‐y* against the DBM genome, was identified as *Pxyellow* and further investigated. This gene was mapped into the region 747 503–755 533 bp in scaffold 25 of the DBM genome. The identified complementary DNA sequence of *Pxyellow* was 1674 bp, containing four exons, each with 277, 186, 1190 and 21 bp in length, and three introns with 4465, 248, and 1650 bp in length, respectively (Fig. 1A).

**Fig. 1:**
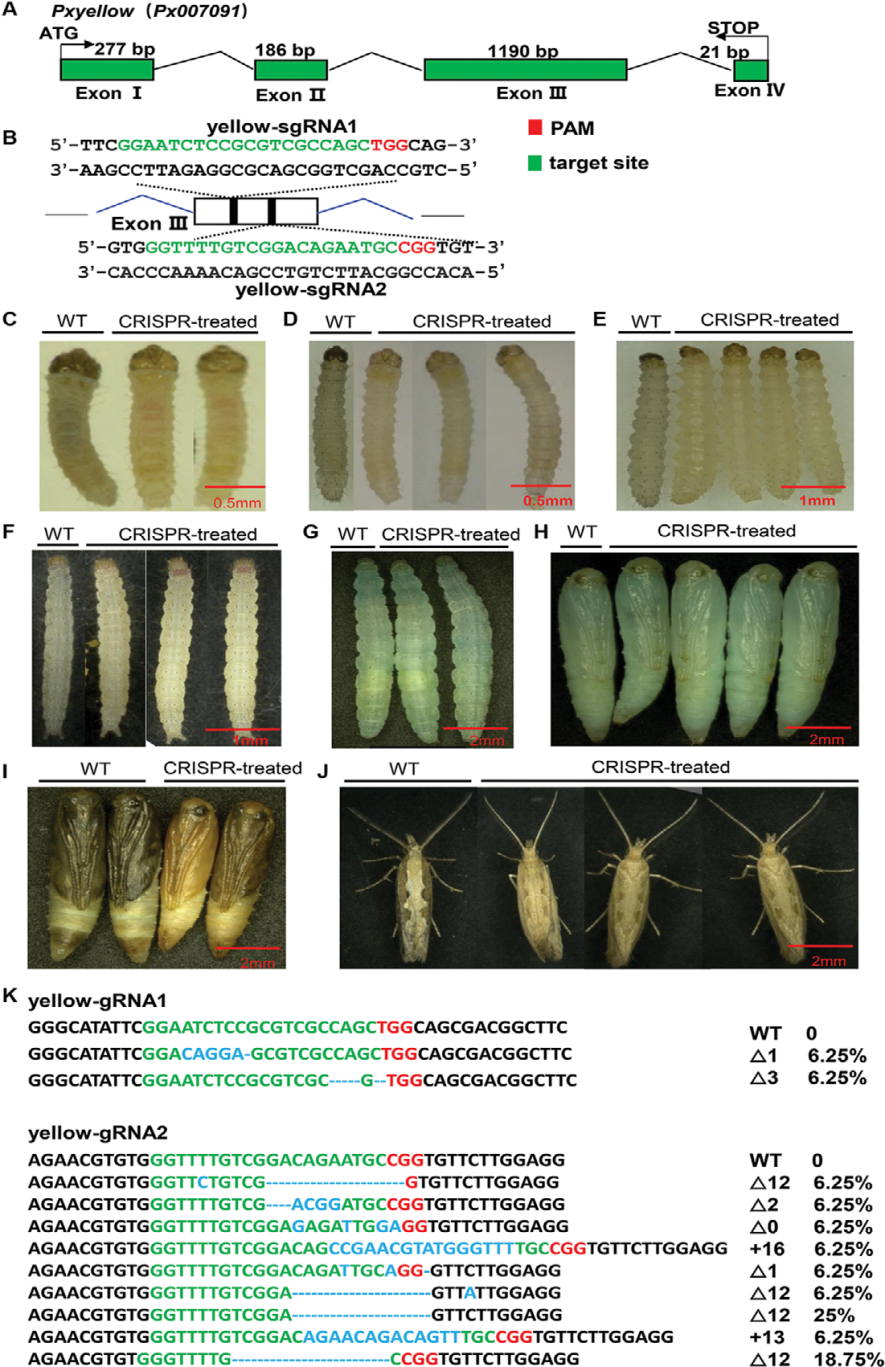
Clustered regularly interspersed palindromic repeats (CRISPR)/CRISPR‐associated protein 9 (Cas9)‐mediated disruption of *Plutella xylostella yellow* ortholog (*Pxyellow*). (A) Gene structure of the *Pxyellow*. *Pxyellow* comprises four exons and three introns. Numbers refer to the lengths in base‐pairs (bp) for each of the exons. ATG and STOP denote the translation initiation and termination codons, respectively. (B) Schematic diagram of CRISPR target sites. The blue broken line indicates intron. The rectangle box represents exon. Two targets site are located in Exon Ⅲ, with an interval of 330 bp. The pigmentation of G_0_ mutant and wild types in different developmental stages, including newly hatched larvae (C), 1st–4th instar larvae (D–G), early‐stage pupae (prepupae) (H), late‐stage pupae (I), and adults (J), are compared. WT: wild type. CRISPR‐treated: *Pxyellow* G_0_ mutants. (K) The representative mutant types in *Pxyellow* G_0_s. The single guide RNA (sgRNA) target sites are highlighted in green, while the pulse amplitude modulation motif is in red, and the insertions and deletions (indels) in blue (Δ: deletions. +: insertions). The numbers of indels are shown at the right. Percentage means mutated clones identified from all analyzed clones.

To introduce CRISPR/Cas9‐mediated mutagenesis in *Pxyellow*, two target sites (yellow‐sgRNA1 and yellow‐sgRNA2) located in Exon III were selected using the ZiFiT Targeter software (Fig. 1B). The off‐target binding capability of two designed single guide RNAs (sgRNAs) was analyzed by blasting target sequences against DBM genome (maximum mismatches = 3). However, no potential off‐target sites were found, indicating a high target specificity of these designed sgRNAs.

In order to generate *Pxyellow* mutants, totally 676 eggs were injected with *Pxyellow*‐sgRNAs and 480 of them hatched, resulting in 71% of hatchability. Additionally, there was no significant difference in hatchability to the negative control (injected with enhanced green fluorescent protein‐sgRNA; hatchability = 71.6%) (Table S3). Based on the observation of yellow pigmentation of 1st instar larvae, 272 of the G_0_ moths were mutated (mutation rate = 57%) (Table S3). Compared with the wild type individuals (with light‐black body and black head capsule), the pigmentation of G_0_ newly hatched larvae (Fig. 1C), 1st (Fig. 1D) and 2nd instar (Fig. 1E) larvae turned yellow (especially apparent change in the color of head capsule), while the body color of treated 3rd instar larvae changed to light yellow (Fig. 1F). The body colors of treated 4th instar larvae (Fig. 1G) and early pupae (Fig. 1H) were not observably different from the color of wild types, while the pigmentation of mutants changed from black or dark brown to yellow/tan in the late pupal stage (Fig. 1I). This indicated that *Pxyellow* was not likely involved in the pigmentation of 4th instar larvae and early pupae, but participated in the melanization of late pupae. A similar result was also obtained from the loss‐of‐function mutant of *Aiyellow‐y* in *A. ipsilon*, showing no significant difference in color between the mutants and wild types in early pupae while the initiation of varied pigmentation occurred in late pupal stage (Chen *et al*., 2018). In addition, the pigmentation of CRISPR‐treated adults in our study changed from gray/black to yellow/tan (Fig. 1J). In total, 33 of G_0_ adults with mutant phenotypes were randomly selected and sequenced, which revealed various insertions or deletions (indels) at both target sites (representative mutant genotypes are provided in Fig. 1K), suggesting the successful mutagenesis in *Pxyellow* locus using the CRISPR/Cas9 system.

To build a homozygous mutant line for further investigation, the G_0_ mutant adults were first crossed with wild type adults in pairs to generate the G_1_ generation. Thirty‐five G_1_ individuals randomly collected from 10 G_0_ parents were sequenced and 11 of them showed mutations (inheritance efficiency = 31.4% [11/35]). In addition, mutant G_1_s were outcrossed with wild types in pairs for producing G_2_ generation, 12 of which were sequenced to confirm their genotypes. Note that both male and female heterozygotes in G_1_ and G_2_ generations showed wild type‐like pigmentation instead of yellowish body color observed in G_0_, indicating that the *Pxyellow* mutation generated here was recessive and *Pxyellow* is not located in sex chromosomes. G_2_ individuals hosting the same mutant type (a 16 bp insertion linked to a frame‐shift mutation; Fig. 1K) were pairwise inbred to generate G_3_s, of which the homozygous mutants showing abnormally yellow pigmentation were maintained as a *Pxyellow* knockout strain (Fig. 2A–D). The phenotype of G_3_ homozygous mutants were mostly consistent with the G_0_ mosaics, although the body color of some G_0_ mosaics retained patchy wild type‐like dark traits while G_3_ homozygotes showed fully yellow pigmentation (Fig. 2D). This observable mutant phenotype in G_0_s was probably linked to the long development time of Lepidoptera, giving more chance for more G_0_ cells to mutate during embryo development. It was noted that the egg color of homozygous mutants at the later embryonic stage was light yellow instead of dark gray observed in their wild type counterparts (Fig. 2A).

**Fig. 2:**
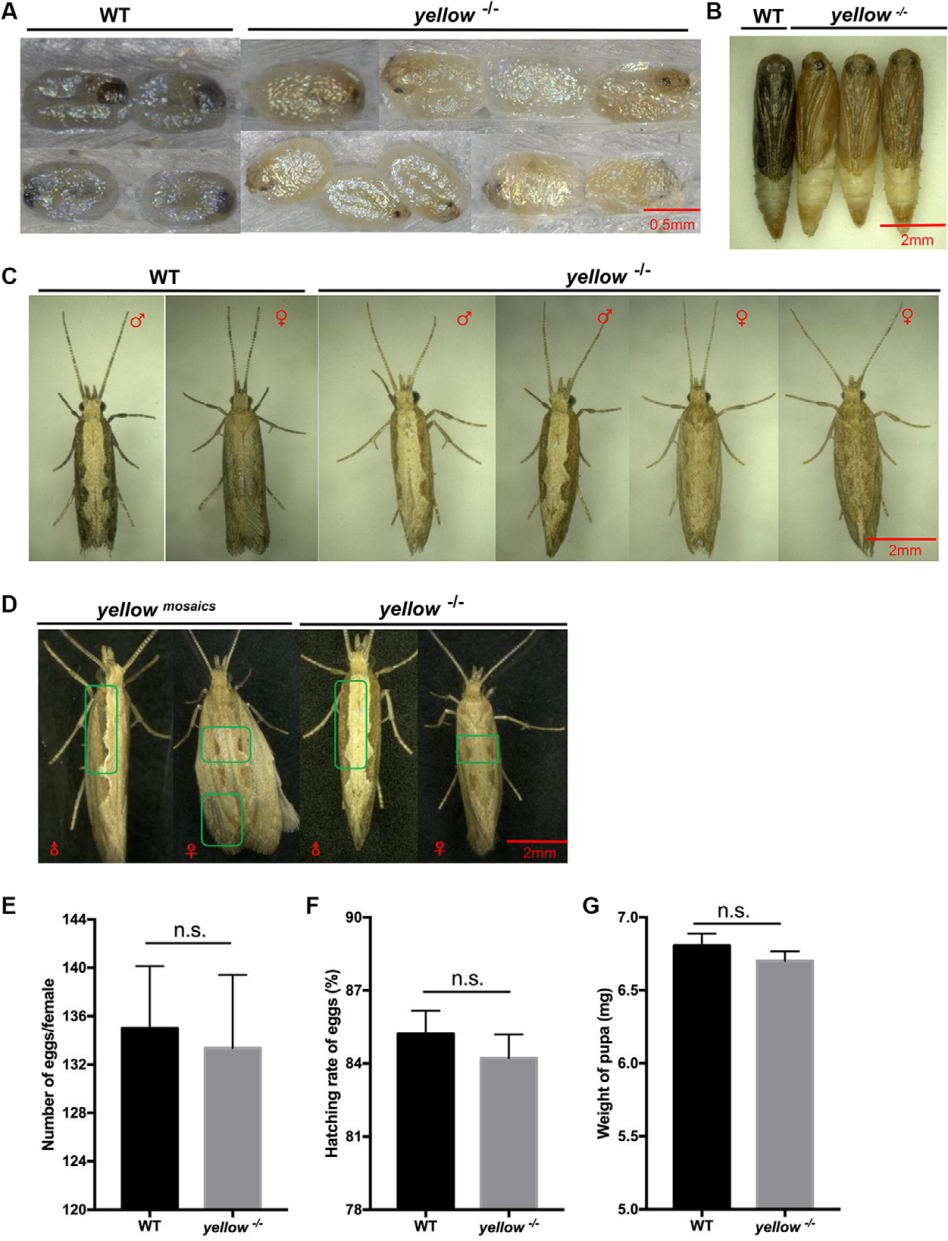
Mutant phenotypes (A–D) and the fitness assay of *Plutella xylostella yellow* ortholog (*Pxyellow*) homozygous knockouts (E–G). Phenotypes of homozygous mutants at egg (A), pupal (B), and adult (C) stages. ♂/♀ symbols represent male/female individuals. (D) Comparison of wing phenotypes between clustered regularly interspersed palindromic repeats (CRISPR)‐treated G_0_ mosaics and homozygous lines. WT: wild type. *yellow ^−/−^*: homozygous mutant. *yellow^mosasic^*: G0 mosaic mutant. Mean number of eggs laid per female (E), mean hatching rate of eggs (F) and mean weight of single pupa (G) are compared between *Pxyellow* mutant line and wild type. The standard error of the mean (±SEM) is shown in figures, and the significance of differences was calculated using the *t*‐test. n.s.: not significant.

To explore whether *Pxyellow* deficiency resulted in any fitness cost in DBM, a series of growth, development and reproduction tests were conducted using both the *Pxyellow* mutant line (group A) and wild type control (group B) with 30 pairs of adults set up in each group. Due to oviposition failure in some replicates (which might be caused by individual fertility variation naturally existing in the DBM population), only 27 pairs of group A and 28 pairs of group B were kept for subsequent fitness analysis. The results showed no significant difference between the *Pxyellow*‐deficient group and the wild type group in oviposition, hatchability or pupal weight (Fig. 2E). These findings suggested that the mutagenesis of *Pxyellow* induced by CRISPR/Cas9 conferred the phenotypic change in body pigmentation without affecting the growth, development or reproduction of DBM. This is consistent with previous research in *A. ipsilon* where deficiency in *Aiyellow‐y* did not obviously affect the moth growth (Chen *et al*., 2018). However, it has been reported that *yellow* family genes comprised rather diverse gene functions. For example, *yellow‐g* and *yellow‐g2*, participated in the development of egg desiccation resistance in *Aedes Albopictus* (Noh *et al*., 2020). Although the egg hatchability was not affected in the *Pxyellow* knockout line, further investigation may be needed to confirm whether it played other roles in DBM embryonic development.

Due to the ease of screening mutant phenotypes, *Pxyellow* can be used as a germline transformation marker for constructing transgenic DBM, providing a useful and measurable tool in genetically based pest control prototypes (i.e., CRISPR/based gene drive systems). Based on our result that disruption of *Pxyellow* likely had no undesirable impact on insect fitness, drivers (e.g., Cas9/sgRNA expressing cassette) can be integrated into *yellow* locus to build viable transgenic lines, followed by cage/field assays to test the spread of transgenics in populations (Gantz & Bier, 2015). It is noted that previous reports in *D. melanogaster* showed changes in male mating behavior and the consequent reduction in male‐specific mating success due to *yellow* null‐mutation (Massey *et al*., 2019). This could be an obstacle in assessing homing efficiency since the transgenics may retain mating disabilities when paired with wild types. Although no observable mating defect was seen in our *Pxyellow* mutant line, a mating competition assay may be required in the future to evaluate the potential ability of mutant lines in transmitting the transgenics into natural populations.

This is the first report of a phenotypic gene, *yellow*, in DBM with CRISPR/Cas9‐mediated loss‐of‐function analysis. In summary, *Pxyellow* played a critical role in the pigmentation patterns in DBM, and the *Pxyellow*‐deficient phenotype could be easily observed through the majority of developmental stages.

## Disclosure

The authors declare they have no competing interests.

## Supporting information


**Fig. S1** Phylogenetic tree of *yellow* gene families based on the alignment of their amino acid sequences from six insect species.

Supporting Materials and methods.

**Table S1** Putative yellow orthologs in *Plutella xylostella*.

**Table S2** GenBank information of yellow sequences used for construction of the phylogenetic tree (Fig. S1).

**Table S3** Mutagenesis mediated by clustered regularly interspersed palindromic repeats (CRISPR)/CRISPR‐associated protein 9 targeted *Pxyellow*.
