# Supplementary material for: CRISPR/Cas9‐based functional analysis of yellow gene in the diamondback moth, Plutella xylostella
Source: Insect Sci. 2020 Sep 18;28(5):1504–9. doi: 10.1111/1744-7917.12870 (PMC8518405; doi:10.1111/1744-7917.12870)
Supplement: Supplementary file 1 — Fig. S1 Phylogenetic tree of yellow gene families based on the alignment of their amino acid sequences from six insect species. [file INS-28-1504-s005.docx]

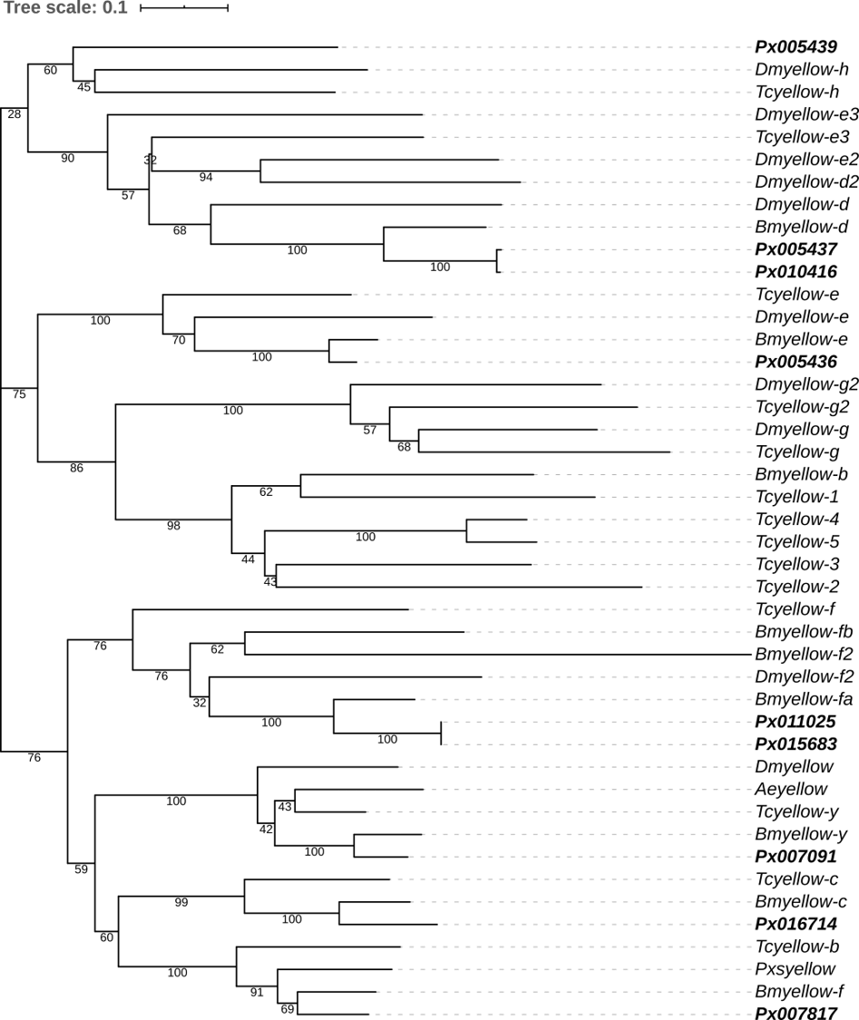


**Fig. S1** Phylogenetic tree of *yellow* gene families based on the alignment of their amino acid sequences from 6 insect species. Neighbor-joining method was conducted to construct the evolutionary tree. The numbers in the middle of branches represent bootstrap values. Putative *P. xylostella* Yellow homologs are marked in bold. Access numbers of sequences used to generate this figure are listed in Table S1 and S2. Abbreviation: *Ae* for *A. aegypti*, *Bm* for *B. mori*, *Dm* for *D. melanogaster*, *Tc* for *T. castaneum*, *Pxs* for *P. xuthus*, *Px* for *P. xylostella*.
