# Supplementary material for: CRISPR/Cas9‐based functional analysis of yellow gene in the diamondback moth, Plutella xylostella
Source: Insect Sci. 2020 Sep 18;28(5):1504–9. doi: 10.1111/1744-7917.12870 (PMC8518405; doi:10.1111/1744-7917.12870)
Supplement: Supplementary file 2 — Supporting Materials and methods. [file INS-28-1504-s002.doc]

**Supporting information**

**Materials and methods**

Experimental P. xylostella strain

The insecticide-susceptible strain of *P. xylostella* was derived from the Institute of Zoology, Chinese Academy of Science (Beijing) and has been maintained in our laboratory since 2015 without exposure to any insecticides. They were reared in a growth chamber at 25 ± 1°C, 50%–70% relative humidity (RH), 16 : 8 h light : dark photoperiod. *P. xylostella* larvae were fed in paper cups with artificial diet containing 20 g yeast powder, 37.5 g raw wheat germ, 1 g vitamin premix, 1 g potassium sorbate, 1 g methyl paraben, 1 g ascorbic acid, 10 g sucrose, 3 g powder of radish seed, 1 mL cola oil, 0.1 mL linoleic acid, 6 g agar and 250 mL water. Adults were allowed to mate in a cage (45 × 45 × 45 cm) and fed with 10% honey solution.

Phylogenetic analysis

*Bombyx mori* yellow/yellow-y protein sequence (NP_001037434.1) (Futahash*i et a*l., 2008) was used as the query against the DBM genome database (http://iae.fafu.edu.cn/DBM/index.php) (Tan*g et a*l., 2014) to identify *yellow* homologs in *P. xylostella* with the BlastP program (Expectation value ≤1E-10). Conserved domain of their amino acid sequences was subsequently confirmed with a NCBI RPS-BLAST program (https://www.ncbi.nlm.nih.gov/Structure/cdd/wrpsb.cgi). The yellow amino acid sequences from 6 insect species (*Aedes aegypti*, *Papilio xuthus*, *Bombyx. mori*, *Drosophila melanogaster*, *Tribolium. castaneum* and *P. xylostella*) were downloaded from NCBI and multiple alignments were completed using MEGA 7 with the ClustalW algorithm. Neighbor-joining method was conducted with 1000 bootstrap replicates to construct the phylogenetic tree. Corresponding access numbers of these sequences available in Genbank were provided in Table S2.

Cloning of Pxyellow

Total RNA was isolated from wildtype *P. xylostella* pupae with TRIzol Reagent (Invitrogen, USA)., after which 500 ng total RNA was used to generate the first-strand cDNA using the Hiscript Reverse Transcriptase (Vazyme Biotech, China) with 1 *μ*L oligo (dT) and 1 *μ*L random hexamers. The *Pxyellow* cDNA was amplified with specific primers (F: 5′-ATG GAG GTT GTA AAA CTA CAA GTT G-3′; R: 5′-CTA AGT CAT TGC CTG CGT TTT-3′). PCR was carried out with Phanta Super-Fidelity DNA Polymerase (Vazyme Biotech, China) under the following conditions: 95 °C for 3 min, and then 34 cycles at 95 °C for 30 s, 55 °C for 30 s, 72 °C for 90 s, and a final elongation step at 72 °C for 10 min. PCR products were isolated from 1% agarose gel. The target band was extracted using an Omega Gel Extraction Kit (Omega, USA) and cloned into pJET1.2 vector (Thermo Fisher Scientific, USA) for sequencing (Biosune, China).

In vitro transcription of Cas9 mRNA and sgRNAs

The sgRNA design and synthesis was conducted referring to previous report (Hwan*g et a*l., 2013). Specifically, two sgRNAs targeting sequences (5′-GGA ATC TCC GCG TCG CCA GCT GG-3′, 5′-GGT TTT GTC GGA CAG AAT GCC GG-3′) were designed with ZiFiT Targeter software by searching Exon III region of *Pxyellow* based on the 5′-GG-N_18_-NGG-3′ rule. The control sgRNA (5′-GGC GAG GGC GAT GCC ACC TA-3′) was used to target the exogenous gene encoding the EGFP protein (Huan*g et a*l., 2017). Off-target sites were predicted by blasting sgRNA target sequences in DBM genome with CRISPR RGEN Tools (http://www.rgenome.net/cas-offinder/). The sgRNAs and Cas9 mRNA were synthesized with a procedure as reported previously (Huan*g et a*l., 2016). In brief, sgRNAs were generated from a ready-to-use vector and subcloned into pJET1.2 vector (Thermo Fisher Scientific, USA) for sequencing. *In vitro* transcription of sgRNAs was completed with MAXIcript T7 kit (Ambion, USA). The PTD1-T7-Cas9 vector (ViewSolid Biotech, China) was linearized with the NotI restriction enzyme (Thermo Fisher Scientific, USA), and the Cas9 mRNA was subsequently synthesized *in vitro* with mMESSAGE mMACHINE T7 Kit (Ambion, USA).

Germline transformation and phenotype analysis

*P. xylostella* adults were kept in a cage allowing them to mate and oviposit on a parafilm sheet covered with cabbage juice. The egg sheet was collected at 15–20 min post oviposition for microinjection. A mixture of 500 ng/*μ*L Cas9 mRNA and 300 ng/μl sgRNAs (i.e.150 ng/*μ*L of each sgRNA) was injected into fresh eggs within one-hour post oviposition and then incubated in the growth chamber described above. Abnormally lighter pigmentation of G_0_s were identified with a stereo microscope (Nikon, SW-2B/22) and the number of mosaics was recorded. G_0_ adults were outcrossed to wildtypes, and the hatching rate of resulting G_1_ eggs was calculated. G_1_ adults were mated to wildtypes again, followed by inbreeding G_2_ individuals containing the same mutant alleles for generating homozygous G_3_ mutants. All the pairs described above were conducted in single pair crossing (i.e. one female adult mated with one male adult). The larvae, pupae, adults and their eggs of G_3_ individuals were screened for mutant phenotypes. To confirm the mutagenesis of the *Pxyellow* locus, genomic DNA of G_0_, G_1_ and G_2_ adults (after mating and oviposition) as well as 4th instar larvae of G_3_ mutants and wildtype control was extracted with DNA Extraction Kit (Omega, USA). A ~500 bp fragment covering the target region was amplified with specific primers (F: 5′-AAC TCG GAT ATG GAC TCA TCG-3′, R: 5′-CAG ACC AAA TTG CGA GTT CG - 3’), and the amplicons were extracted and subcloned for sequencing as described above.

Fitness test

To evaluate fitness effects caused by *Pxyellow* mutation, we conducted comparative tests on the oviposition of adults, the hatchability of eggs, and the weight of pupae between individuals of the homozygous mutants (group A) and the control wildtypes (group B). Adults were individually paired (one male and one female) and reared in the cage as previously described. There were three replicates for each group and ten pairs of adults for each replicate. The resulting data were statistically analyzed with T-test using the GraphPad Pism 6 software.

**References**

Futahashi, R., Sato, J., Meng, Y., Okamoto, S., Daimon, T., Yamamoto, K., Suetsugu, Y., Narukawa, J., Takahashi, H., Banno, Y., Katsuma, S., Shimada, T., Mita, K. and Fujiwara, H. (2008) *yellow* and *ebony* are the responsible genes for the larval color mutants of the silkworm *Bombyx mori*. *Genetics*, 180, 1995–2005.

Huang, Y., Chen, Y., Zeng, B., Wang, Y., James, A.A., Gurr, G.M., Yang, G., Lin, X., Huang, Y. and You, M. (2016) CRISPR/Cas9 mediated knockout of the *abdominal-A* homeotic gene in the global pest, diamondback moth (*Plutella xylostella*). *Insect Biochemistry and Molecular Biology*, 75, 98–106.

Huang, Y., Wang, Y., Zeng, B., Liu, Z., Xu, X., Meng, Q., Huang, Y., Yang, G., Vasseur, L., Gurr, G.M. and You, M. (2017) Functional characterization of Pol III U6 promoters for gene knockdown and knockout in *Plutella xylostella*. *Insect Biochemistry and Molecular Biology*, 89, 71–78.

Hwang, W.Y., Fu, Y., Reyon, D., Maeder, M.L., Tsai, S.Q., Sander, J.D., Peterson, R.T., Yeh, J.R. and Joung, J.K. (2013) Efficient genome editing in zebrafish using a CRISPR-Cas system. *Nature Biotechnology*, 31, 227–229.

Tang, W., Yu, L., He, W., Yang, G., Ke, F., Baxter, S.W., You, S., Douglas, C.J. and You, M. (2014) DBM-DB: the diamondback moth genome database. *Database*, 2014.
