# Supplementary material for: CRISPR/Cas9‐based functional analysis of yellow gene in the diamondback moth, Plutella xylostella
Source: Insect Sci. 2020 Sep 18;28(5):1504–9. doi: 10.1111/1744-7917.12870 (PMC8518405; doi:10.1111/1744-7917.12870)
Supplement: Supplementary file 3 — Table S1 Putative yellow orthologs in Plutella xylostella. [file INS-28-1504-s003.docx]

**Table S1** Putative *yellow* orthologs in *P. xylostella.*

| Gene ID | location | CDS length (bp) | Protein length (aa) | MRJP domain position (aa) |
| --- | --- | --- | --- | --- |
| *Px007091* | scaffold_25: 747503..755533 | 1674 | 559 | 132-416 |
| *Px007817* | scaffold_278: 237645..247724 | 1419 | 473 | 130-416 |
| *Px005439* | scaffold_2: 314999..323208 | 1602 | 534 | 220-508 |
| *Px016714* | scaffold_824: 34607..39582 | 1221 | 407 | 118-405 |
| *Px011025* | scaffold_41: 328180..340162 | 1911 | 637 | 335-625 |
| *Px015683* | scaffold_73: 1509347..1519606 | 1458 | 486 | 240-484 |
| *Px005437* | scaffold_2: 293442..300870 | 1335 | 445 | 119-409 |
| *Px005436* | scaffold_2: 254554..278710 | 1530 | 510 | 111-487 (gap from 255 to 369) |
| *Px010416* | scaffold_382: 52843..57047 | 528 | 176 | 53-174 (truncated) |
