# Supplementary material for: CRISPR/Cas9‐based functional analysis of yellow gene in the diamondback moth, Plutella xylostella
Source: Insect Sci. 2020 Sep 18;28(5):1504–9. doi: 10.1111/1744-7917.12870 (PMC8518405; doi:10.1111/1744-7917.12870)
Supplement: Supplementary file 4 — Table S2 GenBank information of yellow sequences used for construction of the phylogenetic tree (Fig. S1). [file INS-28-1504-s004.docx]

**Table S2** GenBank information of *yellow* sequences used for construction of the phylogenetic tree (Fig. S1).

| Gene | Species | Abbreviation | Access no. |
| --- | --- | --- | --- |
| yellow | *A. aegypti* | *Aeyellow* | XP_001658066.2 |
| yellow-b | *B. mori* | *Bmyellow-b* | AFC87784.1 |
| yellow-c | *B. mori* | *Bmyellow-c* | AFC87785.1 |
| yellow-d | *B. mori* | *Bmyellow-d* | AFC87786.1 |
| yellow-e | *B. mori* | *Bmyellow-e* | AFC87792.1 |
| yellow-f | *B. mori* | *Bmyellow-f* | ALL54586.1 |
| yellow-f2 | *B. mori* | *Bmyellow-f2* | AFC87793.1 |
| yellow-fa | *B. mori* | *Bmyellow-fa* | AFC87787.1 |
| yellow-fb | *B. mori* | *Bmyellow-fb* | NP_001037428.1 |
| yellow-y | *B. mori* | *Bmyellow-y* | NP_001037434.1 |
| yellow | *D. melanogaster* | *Dmyellow* | NP_476792.1 |
| yellow-d | *D. melanogaster* | *Dmyellow-d* | NP_523820.2 |
| yellow-d2 | *D. melanogaster* | *Dmyellow-d2* | NP_611788.1 |
| yellow-e | *D. melanogaster* | *Dmyellow-e* | NP_524344.1 |
| yellow-e2 | *D. melanogaster* | *Dmyellow-e2* | NP_650289.2 |
| yellow-e3 | *D. melanogaster* | *Dmyellow-e3* | NP_650288.1 |
| yellow-f2 | *D. melanogaster* | *Dmyellow-f2* | NP_650247.1 |
| yellow-g | *D. melanogaster* | *Dmyellow-g* | NP_523888.1 |
| yellow-g2 | *D. melanogaster* | *Dmyellow-g2* | NP_647710.1 |
| yellow-h | *D. melanogaster* | *Dmyellow-h* | NP_651912.3 |
| yellow | *P. xuthus* | *Pxsyellow* | KPI99351.1 |
| yellow-1 | *T. castaneum* | *Tcyellow-1* | ACY71064.1 |
| yellow-2 | *T. castaneum* | *Tcyellow-2* | ACY71065.1 |
| yellow-3 | *T. castaneum* | *Tcyellow-3* | NP_001161785.1 |
| yellow-4 | *T. castaneum* | *Tcyellow-4* | NP_001161786.1 |
| yellow-5 | *T. castaneum* | *Tcyellow-5* | ACY71068.1 |
| yellow-b | *T. castaneum* | *Tcyellow-b* | ACY71055.1 |
| yellow-c | *T. castaneum* | *Tcyellow-c* | ACY71056.1 |
| yellow-e | *T. castaneum* | *Tcyellow-e* | ACY71058.1 |
| yellow-e3 | *T. castaneum* | *Tcyellow-e3* | NP_001161913.1 |
| yellow-f | *T. castaneum* | *Tcyellow-f* | ACY71059.1 |
| yellow-g | *T. castaneum* | *Tcyellow-g* | ACY71060.1 |
| yellow-g2 | *T. castaneum* | *Tcyellow-g2* | NP_001161782.1 |
| yellow-h | *T. castaneum* | *Tcyellow-h* | ACY71062.1 |
| yellow-y | *T. castaneum* | *Tcyellow-y* | NP_001161919.1 |
