# Supplementary material for: CRISPR/Cas9‐based functional analysis of yellow gene in the diamondback moth, Plutella xylostella
Source: Insect Sci. 2020 Sep 18;28(5):1504–9. doi: 10.1111/1744-7917.12870 (PMC8518405; doi:10.1111/1744-7917.12870)
Supplement: Supplementary file 5 — Table S3 Mutagenesis mediated by clustered regularly interspersed palindromic repeats (CRISPR)/CRISPR‐associated protein 9 targeted Pxyellow. [file INS-28-1504-s001.docx]

**Table S3** Mutagenesis mediated by CRISPR/Cas9 targeted *Pxyellow.*

| sgRNA | Injected embryos | Hatchability | Mutation of G_0_ |
| --- | --- | --- | --- |
| *Pxyellow*-sgRNA | 676 | 480 (71%) | 272 (57%) |
| EGFP-sgRNA | 380 | 272 (71.6%) | / |
